# Influence of Pneumococcal Conjugate Vaccine on Acute Otitis Media with Severe Middle Ear Inflammation: A Retrospective Multicenter Study

**DOI:** 10.1371/journal.pone.0137546

**Published:** 2015-09-08

**Authors:** Hirotoshi Sugino, Shigeru Tsumura, Masaru Kunimoto, Masuhiro Noda, Daisuke Chikuie, Chieko Noda, Mariko Yamashita, Hiroshi Watanabe, Hidemasa Ishii, Toru Tashiro, Kazuhiro Iwata, Takashi Kono, Kaoru Tsumura, Takahiro Sumiya, Sachio Takeno, Katsuhiro Hirakawa

**Affiliations:** 1 Asa Medical Association, Hiroshima City, Japan; 2 Sugino Pediatric Clinic, Hiroshima City, Japan; 3 Tsumura ENT Clinic, Hiroshima City, Japan; 4 Department of Otolaryngology, Head and Neck Surgery, Graduate School of Biomedical & Health Sciences, Applied Life Sciences, Hiroshima University, Hiroshima City, Japan; 5 Kunimoto ENT Clinic, Hiroshima City, Japan; 6 Noda ENT Clinic, Hiroshima City, Japan; 7 Hiroshima Kyouritsu Hospital, Hiroshima City, Japan; 8 Yamashita Clinic, Hiroshima City, Japan; 9 Watanabe ENT Clinic, Hiroshima City, Japan; 10 Hiroshima City Asa Hospital, Hiroshima City, Japan; 11 Tashiro ENT Clinic, Hiroshima City, Japan; 12 Graduate School of Integrated Arts and Sciences, Hiroshima University, Hiroshima City, Japan; London School of Hygiene and Tropical Medicine, UNITED KINGDOM

## Abstract

The Japanese guidelines for acute otitis media in children recommend classifying acute otitis media by age, manifestations and local findings, and also recommend myringotomy for moderate-grade cases with severe local findings, severe-grade cases, and treatment-resistant cases. The heptavalent pneumococcal conjugate vaccine was released in Japan in February 2010. In Hiroshima City, public funding allowing free inoculation with this vaccine was initiated from January 2011, and the number of vaccinated individuals has since increased dramatically. This study investigated changes in the number of myringotomies performed to treat acute otitis media during the 5-year period from January 2008 to December 2012 at two hospitals and five clinics in the Asa Area of Hiroshima City, Japan. A total of 3,165 myringotomies for acute otitis media were performed. The rate of procedures per child-year performed in <5-year-old children decreased by 29.1% in 2011 and by 25.2% in 2012 compared to the mean rate performed in the 3 years prior to the introduction of public funding. A total of 895 myringotomies were performed for 1-year-old infants. The rate of myringotomies per child-year performed for acute otitis media in 1-year-old infants decreased significantly in the 2 years after the introduction of public funding for heptavalent pneumococcal conjugate vaccine compared to all years before introduction (p<0.000001). Our results suggest a benefit of heptavalent pneumococcal conjugate vaccine for acute otitis media in reducing the financial burden of myringotomy. In addition, this vaccine may help prevent acute otitis media with severe middle ear inflammation in 1-year-old infants.

## Introduction

*Streptococcus pneumoniae* is one of the major causative bacteria for acute otitis media (AOM), which can induce severe illnesses such as mastoiditis, meningitis and epidural abscess. Previous studies have investigated the effectiveness of the heptavalent pneumococcal conjugate vaccine (PCV7) against AOM in terms of changes in AOM episodes and total number of visits to medical institutions made by children. In 2014, an update to a meta-analysis of PCV effects on AOM was reported in the Cochrane collection [1]. One of the authors concluded the PCV7 had modest beneficial effects on healthy infants with a low baseline risk of AOM, according to a meta-analysis of reports by Black et al. [2], Eskola et al. [3], Fireman et al. [4], Kilpi et al. [5], Palmu et al. [6], and O'Brien et al. [7].

Two new pneumococcal conjugate vaccines have been recently released: the 13-valent pneumococcal conjugate vaccine (PCV-13); and the 10-valent pneumococcal non-typable *Haemophilus influenzae* protein D conjugate vaccine (PHiD-CV). Marom et al. reported that a significant reduction in otitis media (OM) visits by children younger than 2 years coincided with the advent of PCV-13 [8], and Ovnat Tamir et al. reported that circulating *S*. *pneumoniae* strains causing "severe" AOM in PCV13-immunized children yielded lower inflammatory responses when compared with unimmunized children [9]. Shiragami et al reported that vaccination with PHiD-CV would result in cost savings from both provider and societal perspectives largely due to reductions in highly prevalent AOM [10].

In Japan, the PCV7 was launched in February 2010 as a vaccination paid out of private expenses. In November 2010, the Japanese government announced an official program recommending PCV7 immunization for <5-year-old children (Provisional Special Fund for the Urgent Promotion of Vaccination). The time to start this program was determined by the respective local governments. In Hiroshima City, PCV7 immunization gained full public funding from January 2011, and the number of vaccinated infants and children subsequently increased markedly. PCV7 was switched to PCV13 in November 2013. We hypothesized that the number of children with AOM showing severe middle ear inflammation and the number of cases requiring myringotomy would both have decreased following the introduction of free PCV7. To determine whether this hypothesis was correct, we conducted a multi-center analysis of the number of myringotomies performed.

The Japan Otological Society, the Japan Society for Pediatric Otorhinolaryngology, and the Japan Society for Infectious Diseases in Otolaryngology released the first edition of clinical practice guidelines for the diagnosis and management of AOM in children in Japan in 2006, with revisions published in 2009 [11] and 2013 [12]. The Japanese guidelines provide criteria for evaluating the degree of AOM and very detailed therapeutic methods using an algorithm based on subsequent severity classification. The first and second guidelines recommend myringotomy for AOM accompanied by severe otoscopic findings and for treatment-resistant cases. Myringotomy is performed after eligibility for the procedure is determined using a surgical microscope or magnifying otoscope. For this reason, changes in the number of myringotomies performed in Japan are thought to reflect changes in the incidence of AOM with severe middle ear inflammation.

We conducted a retrospective study to investigate the number of myringotomies performed to treat AOM with severe middle ear inflammation before and after the introduction of public funding for PCV7, excluding myringotomies for otitis media with effusion (OME) on the medical record and excluding myringotomies with ventilation tube insertion which is performed only for OME and recurrent otitis media. The results from this study were thought to reflect the influence of PCV7 on AOM with severe middle ear inflammation, and thus to clarify the effectiveness of PCV7.

## Methods

Myringotomy cases were retrieved from the electronic database for insurance claim purposes from the participating facilities. All cases were confirmed to have undergone the procedure to treat all-cause AOM (aAOM) upon inquiry at each facility. Each facility submitted data on myringotomies for aAOM, along with sex, age, and month and year of operation in an anonymized form to a central institution. Those data were compiled into a new database created specifically for the present study. The anonymization process was performed in an unlinkable way, so none of the study authors were able to access patient-identifying information. This retrospective study was approved by the ethics committee at Hiroshima City Asa Hospital.

In Asa Area, myringotomy was performed only at specialist otolaryngological facilities. Participating otolaryngological facilities during the study period were both of the hospitals in Asa Area and five otolaryngology clinics. The total number of otolaryngology clinics in Asa Area was 18 in 2008 and 2009, 16 in 2010, and 15 in 2011 and 2012. Percentages of participating facilities (hospitals and clinics) among all facilities were thus 35% in 2008 and 2009, 38.8% in 2010, and 41.2% in 2011 and 2012.

Each facility had used the Japanese guidelines for children under 15 years old [11]. These guidelines provide criteria for evaluating the degree of AOM according to the total scores and recommend myringotomy for AOM of moderate grade with severe otoscopic findings and all cases of severe-grade AOM or treatment-resistant cases, according to the first and second editions of the clinical practice guidelines for the diagnosis and management of AOM in children in Japan (Table 1).

**Table 1:** Comparison of clinical practice guidelines for the diagnosis and management of AOM in children in Japan in 2006, with revisions published in 2009 [11] and 2013 [12].

Edition	First	Second	Third
Year	2006	2009	2013
Age (score)	(0) ≥3 years old > (3)	(0) ≥ 24 months > (3)	(0) ≥ 24 months > (3)
Score	mild, moderate, severe	mild, moderate, severe	mild, moderate, severe
**Clinical manifestations**			
**Fever**	37°C ≤T<38°C (0,1,2)	37.5°C≤T<38.5°C(0,1,2)	37.5°C≤T<38.5°C (0,1,2)
**Ear pain**	0, 1, 2	0, 1, 2	0, 1, 2
**Crying/bad temper**	0 or 1	0 or 1	0 or 1
**Otoscopic findings**			
**Hyperemia**	0, 2, 4	0, 2, 4	0, 2, 4
**protrusion**	0, 4, 8	0, 4, 8	0, 4, 8
**Otorrhea**	0, 4, 8	0, 4, 8	0, 4, 8
**Light reflex**	-	0 or 4	-
**Classification of severity of AOM according to the total scores**			
**Mild**	5≤	9≤	5≤
**Moderate**	6≤ ≤11	10≤ ≤15	6≤ ≤11
**Severe**	12≤	16≤	12≤

The main difference between the first and second guidelines is the recommendation for duration of antimicrobial use. All guidelines recommend myringotomy for all AOM with severe otoscopic findings. The first and second guidelines recommend second myringotomy for all treatment-resistant AOM, and the third recommends myringotomy on a case-by case basis. Indications for myringotomy are the same in the first and second editions.

The total number of PCV7 immunizations in Hiroshima City during this period was provided by the Health Services and Welfare Division of Hiroshima City. Changes in the population of Asa Area were examined using the Hiroshima City Statistic Reports. In addition, infection status during this period, including influenza, mumps, respiratory syncytial virus (RSV) infection, mycoplasma, and pharyngoconjunctival fever were assessed using data from the Hiroshima City Infectious Disease Surveillance Center.

### Patients

In Hiroshima City, public funding for PCV7 immunization was initiated in January 2011. Typically, infants were vaccinated four times: three times before the age of 12 months, and once at 12–15 months as a booster immunization. In 2011 and 2012, due to the government policy on emergency vaccination promotion, unvaccinated 7- to <12-month-old infants were vaccinated three times, 12- to <24-month-old children were vaccinated twice, and 24- to <60-month-old children were also vaccinated once. According to information from the Health Services and Welfare Division of Hiroshima City, the total number of children eligible for PCV7 immunization in Hiroshima City was approximately 11,000 (approximately 4,000 in Asa Area) for each age group. In March 2012, estimated inoculation rate for PCV7 administered for the entirety of Hiroshima City were 100% for those born in 2011, 76% for 2010, 69% for 2009, 53% for 2008, and 53% for 2007. Since these estimates do not include children who received the vaccination paid out of private expenses, actual rates of child vaccination were assumed to be greater than those listed above.

We investigated all patients with myringotomies performed for aAOM at two hospitals and five clinics in Asa Area of Hiroshima City during the 5-year period from January 2008 to December 2012.

### Statistical analysis

Using age-group populations of Asa Area as parameters, the chi-square test was performed to determine whether differences existed in the number of myringotomies between fiscal years in each age group. If significant differences were observed, Ryan's multiple comparison tests were applied to determine which pair of years showed the significant difference.

## Results

**Fig 1:**
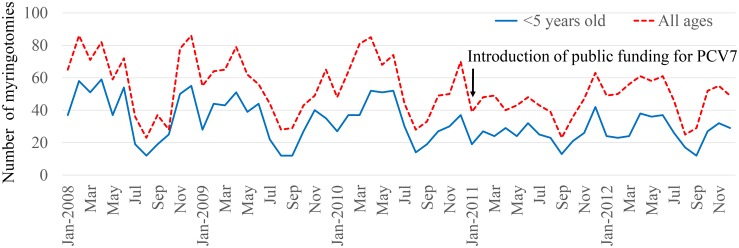
Incidences of myringotomy for all ages (dotted line) and under 5 years old, according to year. Arrow (↓) indicates the introduction of public funding for PCV7. Total number of cases is 3,165 (male, 49%; female, 51%), with 1,916 cases under 5 years old.

**Fig 2:**
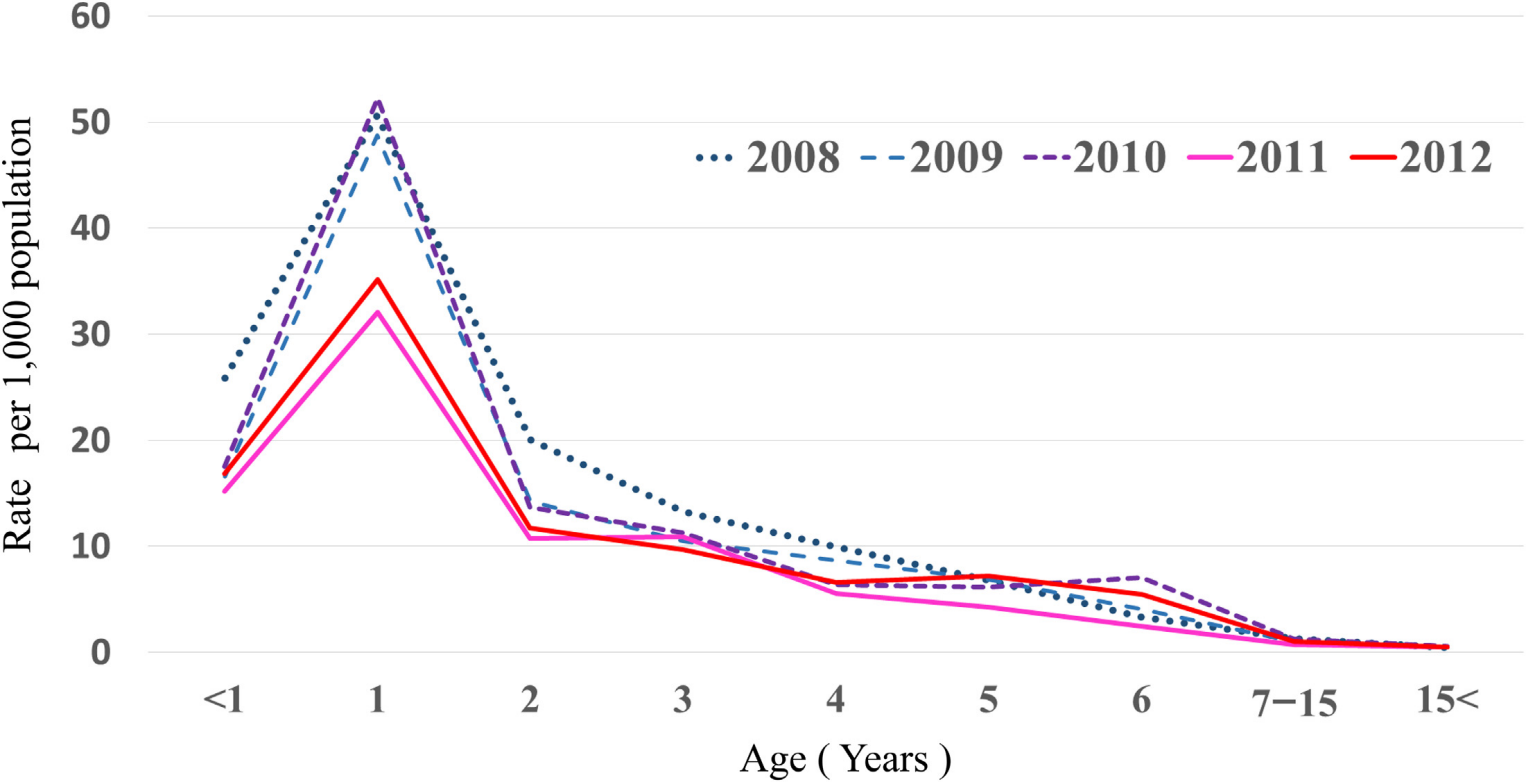
Rate of myringotomy for acute otitis media per 1,000 population-years in age groups, according to year. Public funding for PCV7 was introduced in January 2011.

**Fig 3:**
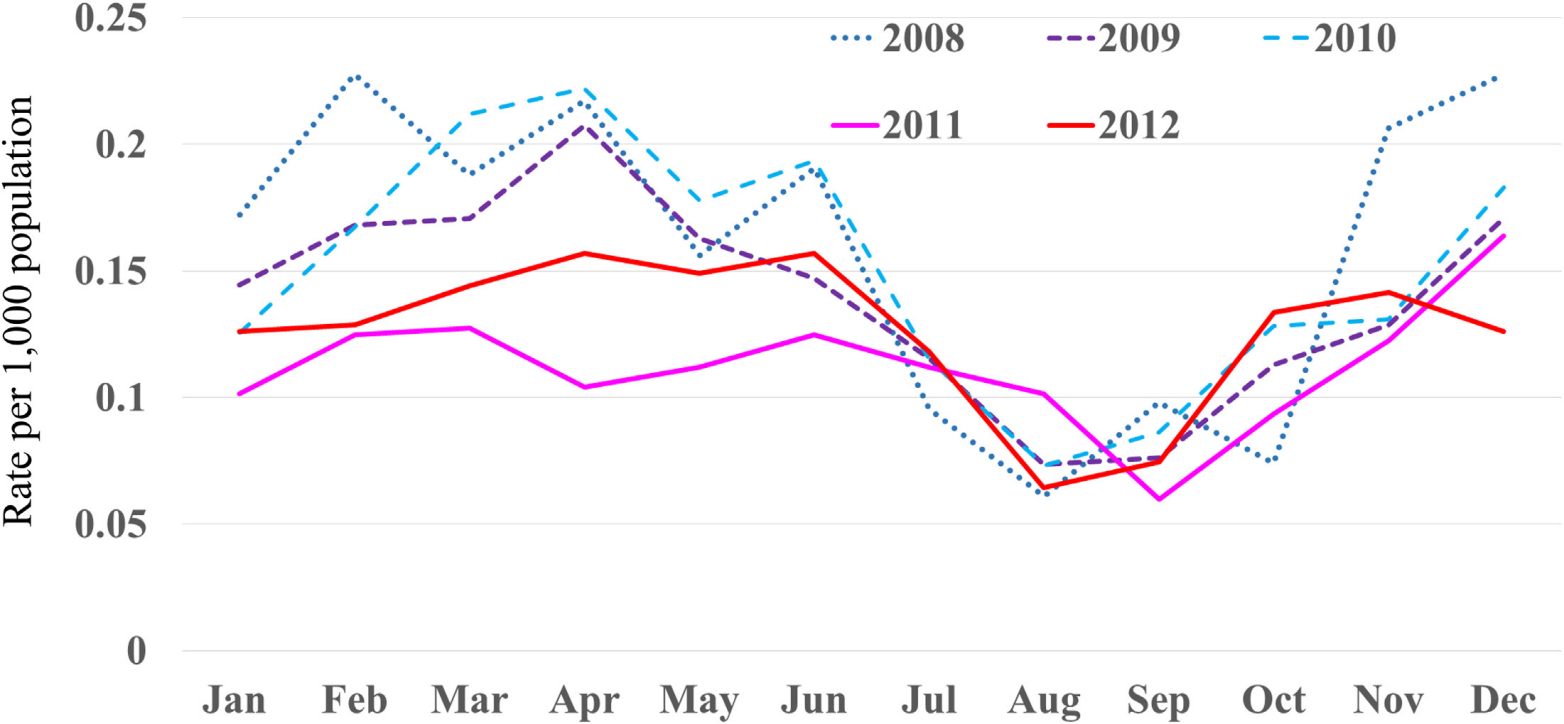
Seasonal changes in rate of myringotomy for acute otitis media per 1,000 population according to year. Public funding for PCV7 was introduced in January 2011.

Myringotomy for aAOM was performed in a total of 3,165 cases of children and adults during the study period (49% males, 51% females), of whom 1,916 were <5 years old. In addition, 1,540 patients were ≤2 years old, and 895 patients were 1 year old. The number of myringotomies performed after the introduction of PCV7 funding decreased during most months, and these reductions were notable in children <5 years old (Fig 1). Analysis by age group showed a marked reduction in rate of myringotomies among 1-year-old infants (Fig 2). Little change was seen between before and after introduction in ≥5-year-old children and adults. Rates of myringotomy per child-years in ≤2-year-olds were reduced by 33.2% (2011) and 26.3% (2012) compared to the mean number during the 3 years before PCV7 introduction. Rates of myringotomy in <5-year-olds were reduced by 29.1% in 2011 and 25.2% in 2012 compared to the mean number during the 3 years before free PCV7 introduction. For changes in each month, other than July, August, and December, all months showed either a decreased or similar rate of myringotomies after free PCV7 introduction compared to before introduction (Fig 3).

Changes in the target population were investigated. During the study period, the mean total population of Asa Area was 382,000. The increase in total population compared to 2008 was +1.6% in 2011 and +2.9% in 2012.

With the population of Asa Area by age as a parameter, chi-squared tests were performed to determine whether differences in the number of myringotomies existed between fiscal years in each age group. At the 0.05 significance level, significant differences were identified for 0 years old, 1 year old, 2 years old, and 6 years old, with p-values of 0.003781, 0.000001>, 0.004885, and 0.01721, respectively. No significant differences were seen for 3, 4, or 5 years old (Table 2).

**Table 2:** Statistical analysis of the number of myringotomies performed before and after introduction of the free heptavalent pneumococcal conjugate vaccine using the chi-square test.

Age	Number of myringotomies / population					*P*
(years)	2008	2009	2010	2011	2012	
<1	**99** / 3,828	67 / 4,044	*70* / 3,995	*61* / 4,005	*63* / 3,742	0.003781
1	**207** / 4,086	**195** / 3,997	**216** / 4,132	*132* / 4,116	*145* / 4,120	<0.000001
2	**77** / 3,840	59 / 4,131	55 / 4,016	*45* / 4,181	*49* / 4,167	0.004885
3	53 / 4,000	41 / 3,914	47 / 4,167	44 / 4,034	41 / 4,212	0.6338
4	40 / 4,021	35 / 4,022	25 / 3,927	23 / 4,158	27 / 4,076	0.09817
5	28 / 4,131	28 / 4,093	25 / 4,059	17 / 3,970	30 / 4,165	0.4789
6	14 / 4,196	17 / 4,163	29 / 4,117	10 / 4,053	22 / 4,022	0.01721

The bold numbers mean significant differences were identified.

Ryan's multiple comparison test was used to determine between which fiscal years differences in the number of myringotomies existed for each age group showing significance according to chi-squared testing (0-, 1-, 2-, and 6-year-olds). The number of myringotomies performed in 1-year-old children was significantly lower in 2011 and 2012 compared to 2008, 2009, and 2010. In 0- and 2-year-old children, numbers of myringotomies were significantly lower in 2011 and 2012 compared to 2008 (Table 3). For 6-year-old children, the number of myringotomies was significantly lower in 2011 than in 2010.

**Table 3:** Statistical analysis of significance between fiscal years using Ryan’s multiple comparisons for the number of myringotomies performed before and after introduction of the free heptavalent pneumococcal conjugate vaccine.

<1 year old		1 year old		2 years old	
Period	*P* (#)	Period	*P* (#)	Period	*P* (#)
2008–2009	0.00264 (0.00667)	2008–2011	0.00002 (0.0067)	2008–2011	0.00041 (0.005)
2008–2010	0.01120 (0.02)	2009–2011	0.00009 (0.01)	2008–2012	0.00218 (0.00667)
2008–2011	0.00046 (0.005)	2010–2011	0.00001 (0.005)		
2008–2012	0.00510 (0.01)	2008–2012	0.00071 (0.01)	**6 years old**	
		2009–2012	0.00224 (0.02)	Years	*P* (#)
		2010–2012	0.00024 (0.00667)	2010–2011	0.00195 (0.005)

^#^ nominal level of significance.

**Fig 4:**
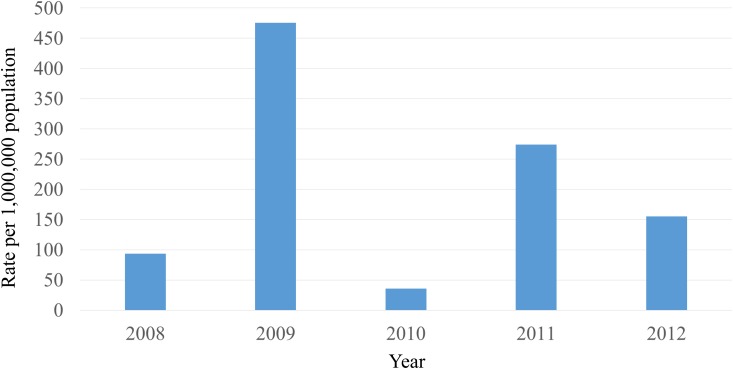
Rate of the number of reports per fixed point of influenza as ratio per 1 million population according to year from Hiroshima City Infectious Disease Surveillance Center. Public funding for PCV7 was introduced in January 2011.

The trend in infections other than *S*. *pneumoniae* infections was investigated. In 2011 and 2012, the Hiroshima City Infectious Disease Surveillance Center Data did not show marked reductions in the number of reports per fixed point of influenza in the ratio per ahead of population (Fig 4). Weekly reports from patient surveillance by The Hiroshima City Infectious Disease Surveillance Center did not show any marked reductions in mumps, mycoplasma, pharyngoconjunctival fever, or RSV infection after the introduction of free PCV in 2011 and 2012.

## Discussion

In 2011 and 2012, compared to the mean number of myringotomies performed in the 3 years before the initiation of free PCV7 immunization by Hiroshima City, the rate of myringotomies performed for aAOM in ≤2-year-old children decreased by 33.2% and 26.3%, respectively. While data were not limited to AOM caused by *S*. *pneumoniae*, statistical analysis showed that the rate of myringotomies in 1-year-old infants decreased significantly after introduction of public funding for PCV7 in all years compared to before introduction. Similar results were shown by a facility that submitted data that included numbers from February 2009, and in ≤5-year-old children rate of myringotomy for aAOM divided by 1,000 child-years were 2.98, 3.56, 0.98, and 1.08 for 2009, 2010, 2011, and 2012, respectively, although these were not added to the present investigation.

The fourth Japanese nationwide survey of clinical isolates from patients with otolaryngological field infections in 2007 reported the detection rate of *S*. *pneumoniae* for AOM was approximately 34% in 2007 [13], and the detection rate of the fifth investigation in 2012 was approximately 29.2%[14]. Kamiya et al. [15] reported the detection rate of *S*. *pneumoniae* as approximately 31.7% from 2005 to 2006, while Otsuka et al. [16] reported a rate of 37%. According to epidemiological surveillance conducted by Chiba et al. [17], serotypes of the PCV7 cover 75.4% of strains isolated from children with invasive pneumococcal disease. In addition, Hotomi et al. reported that serotypes of the PCV7 covered 60.6% of strains isolated from middle ear fluid samples in cases of AOM from 2006 to 2007 [18]. Kitamura et al. described in clinical practice guidelines for AOM that PCV7 covers 62.9% of serotype and 78.0% of drug-resistant *S*. *pneumoniae* isolated from middle ear effusions of children with AOM and a preventive effect of 34.4–62.5% against *S*. *pneumoniae* and 39.8–49.8% against drug-resistant *S*. *pneumoniae* was expected [12]. From these findings, ignoring the *S*. *pneumoniae* serotype that causes cross-reaction, the calculated effect of PCV7 introduction on AOM was thought to be about 18–23%. The rate of reduction since February 2011 was greater than predicted from the serotype and detection rate of *S*. *pneumoniae* in AOM. These findings may be due to the stronger pathogenicity of *S*. *pneumoniae* compared to non-typable *H*. *influenzae* or *M*. *catarrhalis*, increasing the susceptibility to severe middle ear inflammation.

To investigate decreases in the rate of myringotomy performed, changes in population, and changes in viral or other bacterial infections prevalent in that area, the release of new antimicrobials and treatment outcomes must first be accounted for.

The population of Asa Area gradually increased between 2008 and 2012. In addition, no particular changes in the regional prevalence of known respiratory viral infections or other bacterial infections were seen in 2011 or 2012. Moreover, the months in which the number of myringotomies decreased in <5-year-olds were January through June and November in both 2011 and 2012, and this time frame was considered to be too long to represent an effect of any prevalence of specific infections, and the age was also restrictive.

Tebipenem pivoxil (TBPM) was released in August 2009 and tosufloxacin (TFLX) dry syrup was released in January 2010 with approval for coverage by the Japanese public health insurance for use in the treatment of severe AOM. Those antimicrobials have been recommended for severe cases since the third edition of the Japanese guidelines for AOM, published in 2013 [12]. In that third edition, second myringotomy was no longer recommended for all cases of AOM with severe middle ear inflammation and one of those antimicrobials was recommended for AOM with severe middle ear inflammation on a case-by-case basis without myringotomy. However, these third edition recommendations were not in force during the period of the present investigation. Yamanaka stated that, based on the Japan Medical Data Center Claims Database for neonates to children, while adoption of PCV7 vaccination may not have had a strong influence on the incidence of AOM or myringotomy rate in Japan, wider use of the newly developed antimicrobials TFLX and TBPM appears to have played an important role in the decline of myringotomy rate over time [19]. Controlling for the effects of these new antimicrobial on the reduced number of myringotomies is difficult, and virtually no data concerning the safety of TFLX in <1-year-olds and of TBPM in <1- month-olds are available. This is because the release of powerful new antimicrobial may have caused physicians to hesitate in performing surgeries for ambiguous cases. We suspect that PCV7 was most likely included, for the following reasons. First, no rapid increase in the usage of these antimicrobial in Hiroshima City or Hiroshima Prefecture was identifiable immediately after January 2011. Second, decreases in myringotomies among <1-year-old infants are not explicable according to use of TFLX, since this age group is contraindicated for use of TFLX. Third, since 2011, the number of myringotomies has remained unchanged in ≥5-year-old children and adults, who are able to take other strong new antimicrobials.

This study is the first to evaluate changes in the severity of aAOM and the evaluation used the number of myringotomies performed in accordance with the Japanese guideline [11]. While meta-analysis of the effectiveness of PCV7 in AOM has shown PCV7 to only have slight beneficial effects or some protective effect in healthy infants based on frequency comparisons using episodes and numbers of consultations at medical institutions concerning AOM [1, 20], our results suggest a benefit of PCV7 for AOM in reducing the financial burden of myringotomy. In addition, this vaccine may help prevent AOM with severe middle ear inflammation in 1-year-old infants. Even with careful consideration, based on the fact that free PCV7 immunization for children in Hiroshima City began in January 2011, it seems natural to reason that PCV7 represents an extremely important factor in the decreased incidence of middle ear inflammation among <5-year-old children.
